# Helicopter emergency medical service for time critical interfacility transfers of patients with cardiovascular emergencies

**DOI:** 10.1186/s13049-021-00981-4

**Published:** 2021-12-07

**Authors:** Lorenz Meuli, Alexander Zimmermann, Anna-Leonie Menges, Mario Tissi, Stefan Becker, Roland Albrecht, Urs Pietsch

**Affiliations:** 1 Department of Vascular Surgery, University Hospital Zurich, Raemistrasse 100, CH-8006 Zurich, Switzerland; 2 Swiss Air-Ambulance, Rega (Rettungsflugwacht/Guarde Aérienne), Swiss Air-Rescue, Zurich, Switzerland; 3 Department of Anesthesiology and Intensive Care Medicine, Cantonal Hospital St.Gallen, St. Gallen, Switzerland; 4 Department of Emergency Medicine, Inselspital, Bern University Hospital, University of Bern, Bern, Switzerland

**Keywords:** Centralisation, Interfacility transfers, Cardiovascular emergencies, Helicopter emergency medical service, HEMS, Ground emergency medical service, GEMS, rAAA, Stroke, Myocardial infarction

## Abstract

**Background:**

The goal of improving quality through centralisation of specialised medical services must be balanced against potential harm caused by delayed access to emergency treatments in rural areas. This study aims to assess the duration of transfers of critically ill patients with cardiovascular emergencies from smaller hospitals to major medical centres by a helicopter emergency medical service (HEMS) in Switzerland.

**Methods:**

This retrospective observational cohort study includes all consecutive emergency interfacility transfers (IFTs) conducted by Switzerland’s largest HEMS provider between July 3rd, 2019, and March 31st, 2021. All patients with acute myocardial infarction, non-traumatic strokes, ruptured aortic aneurysms, and other acute vascular emergencies were included. The duration and distance of each HEMS IFT were compared to calculated distances and duration of travel for the same missions using ground-based transportation (GEMS). The ground-based mission distance beyond which the total mission duration of HEMS is expected to be faster than GEMS was calculated.

**Findings:**

A total of 645 patients were transferred for stroke (n = 364), myocardial infarction (n = 252) and other acute vascular emergencies (n = 29). The median total mission duration from emergency call to landing at the destination was 59.9 (IQR 51.5 to 70.5) minutes. The median road distance for the same missions was 60 (IQR 43 to 72) km. Regression analysis revealed that HEMS is expected to be faster if the road distance is more than 51.3 km.

**Interpretation:**

Centralisation of specialised medical services should be accompanied by a comprehensive and specialised rescue chain. HEMS in Switzerland ensures time-sensitive IFT in medical emergencies, even in topographically challenging terrain.

**Graphical Abstract:**

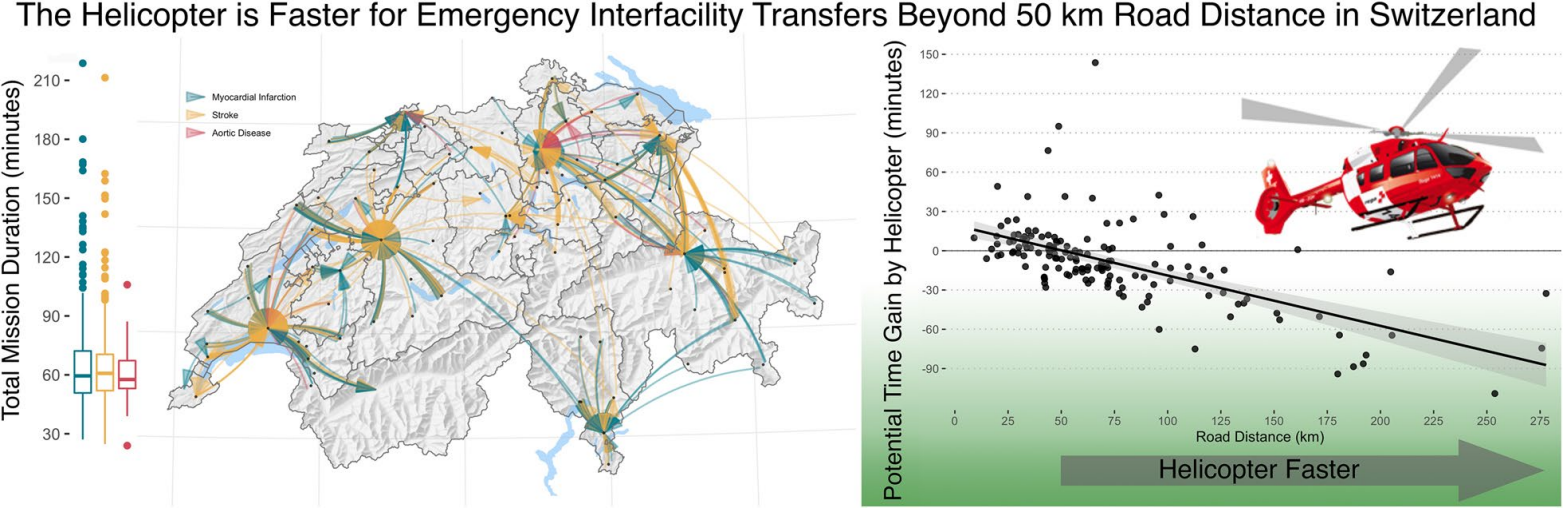

## Background

Centralisation of specialised medical services improves treatment outcomes [1, 2]. This has been demonstrated for planned oncological surgery as well as for treatment in emergency situations such as ruptured abdominal aortic aneurysms (rAAA) [3–5]. However, the centralisation of medical services in larger, more specialised facilities will inevitably lead to medical service deprivation in rural areas. Therefore, the goal of improving quality through centralisation of specialised medical services must be weighed against the potential harm caused by delayed access to emergency treatments.

Time to treatment is especially important in cardiovascular emergencies, where every delay is directly associated with morbidity and mortality [7, 8]. However, current treatment guidelines recommend that patients with myocardial infarctions, strokes, or acute limb ischaemia be transferred to specialised centres capable of performing interventional revascularisation [7–9]. For the management of rAAA, it has been demonstrated that the outcomes of patients transferred to well-equipped centres were not different from those of patients presenting directly at the centre that performed the aneurysm repair [10]. In the UK, this has led to a consensus among vascular specialists that patients with rAAA should be transferred to vascular centres even if they show impaired consciousness and require inotropes [11]. Further, a recently published Vascunet study in 11 European countries, demonstrated that centralisation is associated with better outcomes in aortic surgery [6].

Current treatment guidelines for the management of ST-elevation myocardial infarction recommend the implementation of strategies to facilitate the transfer of patients with cardiovascular emergencies to major medical centres [8, 9, 12]. Thus, a specialised rescue chain with short response time should be available 24/7 and total mission times should be minimised. However, there is inconsistent data in the literature on the efficiency and added clinical benefits of helicopter emergency medical services (HEMS) for interfacility patient transfer (IFT) compared to ground emergency medical service (GEMS) transport [13–16]. Up today, only three studies compared HEMS with GEMS for IFT of patients with medical emergencies. Two articles included trauma patients only and demonstrated that HEMS was significantly faster than GEMS but could not identify a distance threshold beyond which helicopter transport was faster [17–19]. Svenson et. al compared interfacility transfers in Wisconsin and concluded that HEMS is generally faster, but ambulance would be similarly fast if the response and dispatch time were minimized [19].

This study aims to assess the duration of HEMS transfers of patients with cardiovascular emergencies from smaller regional hospitals to major hospitals in Switzerland. In Switzerland, the Alps pose topographical as well as meteorological challenges that can hinder access by GEMS as well as HEMS operating under visual flight rules. In fact, the Alps cover about 58% of the country and roughly 23% of Switzerland’s surface is over 2000 m above sea level. Still, approximately 11% of the Swiss population lives there and this minority should not be deprived of medical services [20]. Further, the federalistic organisation of GEMS might also influence the availability of personnel for inter-regional transportation. Comparison with the estimated duration of ground-based transport for the same transfers should help physicians decide whether to request HEMS or GEMS support in time-critical cardiovascular emergencies.

## Methods

### HEMS in Switzerland

In Switzerland, four organisations provide 24/7 physician-staffed HEMS capable of pre-hospital retrievals and IFT. Rega, the largest of these organisations, responds to emergencies in all regions of Switzerland, operating 18 helicopters at 12 bases. These are distributed throughout the country so that any location within the base’s operational area can be reached at any time within 15 min after alert.

The Rega HEMS crew comprises a pilot, a paramedic and an emergency physician. HEMS physicians require board certification in anaesthesiology and certification in pre-hospital emergency medicine. Rega operates independently of hospitals and conducts in Switzerland around 12,000 HEMS operations annually. Approximately one-third of these missions are secondary missions (i.e. IFTs). In contrast, GEMS is often organized by hospitals and operates preferably local primary missions. Since hospitals want to keep GEMS emergency physicians available for these primary missions, GEMS is scantily available to conduct emergency IFTs. Therefore, HEMS providers are regularly asked to perform these IFT missions.

The helicopters are all equipped with avionics that permit night operations with and without night vision goggles under visual flight rules, but also under instrument flight rules to provide fast advanced prehospital emergency medical care and IFT even under conditions with impaired visibility.

### Study design and participants

This retrospective observational cohort study includes all consecutive HEMS IFT conducted by Rega between July 3rd, 2019, and March 31st, 2021.

Details of every operation were prospectively entered into an SAP database including aviation information such as time, duration, and geographic positions, as well as patient characteristics such as age, sex, and diagnosis. The diagnoses were made by physicians at the referring hospitals and coded in accordance with the 10th version of the International Classification of Diseases (ICD-10).

The study included all consecutive patients transferred by Rega for acute myocardial infarction (I21), acute non-traumatic stroke (I60, I61, I62), aortic dissection (I71.04–07), ruptured aortic aneurysm (I71.1, I71.3, I71.5, I71.8), as well as arterial thrombosis and embolization (I74) to major hospitals. Missions for elective transfer of patients with the same ICD codes back to regional hospitals after emergent treatment were excluded.

The local ethics committee of St. Gallen (EKOS) granted permission to use the anonymised patient data of all HEMS operations carried out within the requested period (EKOS St. Gallen 15.04.2021, BASEC Nr. 2021–00,416 EKOS 21/064). Individual consent of the anonymized participants was waived. This study is reported in accordance with the STROBE statement [21].

### Definitions and statistics

Mission characteristics were analysed regarding mission duration, mission distance, time of day, and diagnosis of the transferred patients. The response time was calculated from emergency call to landing at the referring institution. The total mission time was calculated from emergency call to landing at the destination institution. Night-time was defined according to Commission Regulation (EU) Nr. 965/2012 (commercial air transport operations regulation) as the period between the end of evening civil twilight and the beginning of morning civil twilight.

Patients were grouped under cardiac diseases, strokes, or vascular diseases according to the ICD-10 codes. Institutions were grouped according to the Swiss Federal Office of Public Health classification for hospitals [22]. Institution codes include the two main groups “general hospitals” and “specialty clinics”. General hospitals include five levels of care where level I and II indicate university hospitals and other tertiary referral centers (i.e. "major hospitals"), whereas level III to V indicate smaller hospitals for secondary care (i.e. "regional hospitals"). Of note, several level II hospitals are quite small and do not offer the entire specialised medical service required to treat patients with acute cardiovascular diseases. Those hospitals are organised in groups and the groups are categorised as level II even if the specific institution does not necessarily fulfil the criteria for level II itself. Specialty clinics include surgery, psychiatry, rehabilitation, geriatrics, gynaecology paediatrics, and other medical specialities [22].

To explore the efficiency of HEMS, geographical plotting with analysis of the direct distance between hospitals was performed. Due to the topographical challenges that can hinder GEMS in the mountains, a topographic relief was visualised using swisstopo data from 2016 [20]. Further, distance and duration of travel for ground-based transportation were calculated for all missions using the R package "gmapsdistance" with the Google Maps Distance Matrix interface [23]. A reasonable general preparation and handover duration of 10 min (dispatch time) was added to every mission. The calculated distances and travel times for GEMS were compared with the actual HEMS total mission durations. A univariate linear regression model was built to determine ground-based mission distance equivalent to mission duration reported for HEMS and GEMS.

Continuous variables were summarised by mean ± standard deviation if normally distributed or by median and interquartile range (IQR) if skewed. Normality was tested using the Shapiro-Wilks test. Categorical variables were summarised with counts and percentages for each level of the variable. Due to the low number of missing data (< 1%) a complete case analysis was performed without data imputation. All analyses were done with R-Studio, version 3.6.3, on MacOS version 10.15.7 [24]. All p-values are two-sided with an alpha-level of 5%.

## Results

Between July 3rd, 2019, and March 31st, 2021, Rega’s national emergency coordination centre received a total of 4,985 requests for IFT. Of these requests, 366 (7.3%) had to be declined due to poor weather conditions. One hundred forty-eight (2.9%) were cancelled by the requesting hospital, and 94 (1.9%) were not conducted for unknown reasons. Of the 4,377 missions that were started, 4,283 (97.9%) were completed. 3,636 (83.1%) missions involved patients transferred for reasons other than cardiovascular or cerebral emergencies, and two (0.05%) international missions were excluded. The final study cohort comprised 645 patients who were treated for acute cerebrovascular insults (n = 364), acute myocardial infarction (n = 252) and other acute vascular emergencies (n = 29).

**Fig. 1:**
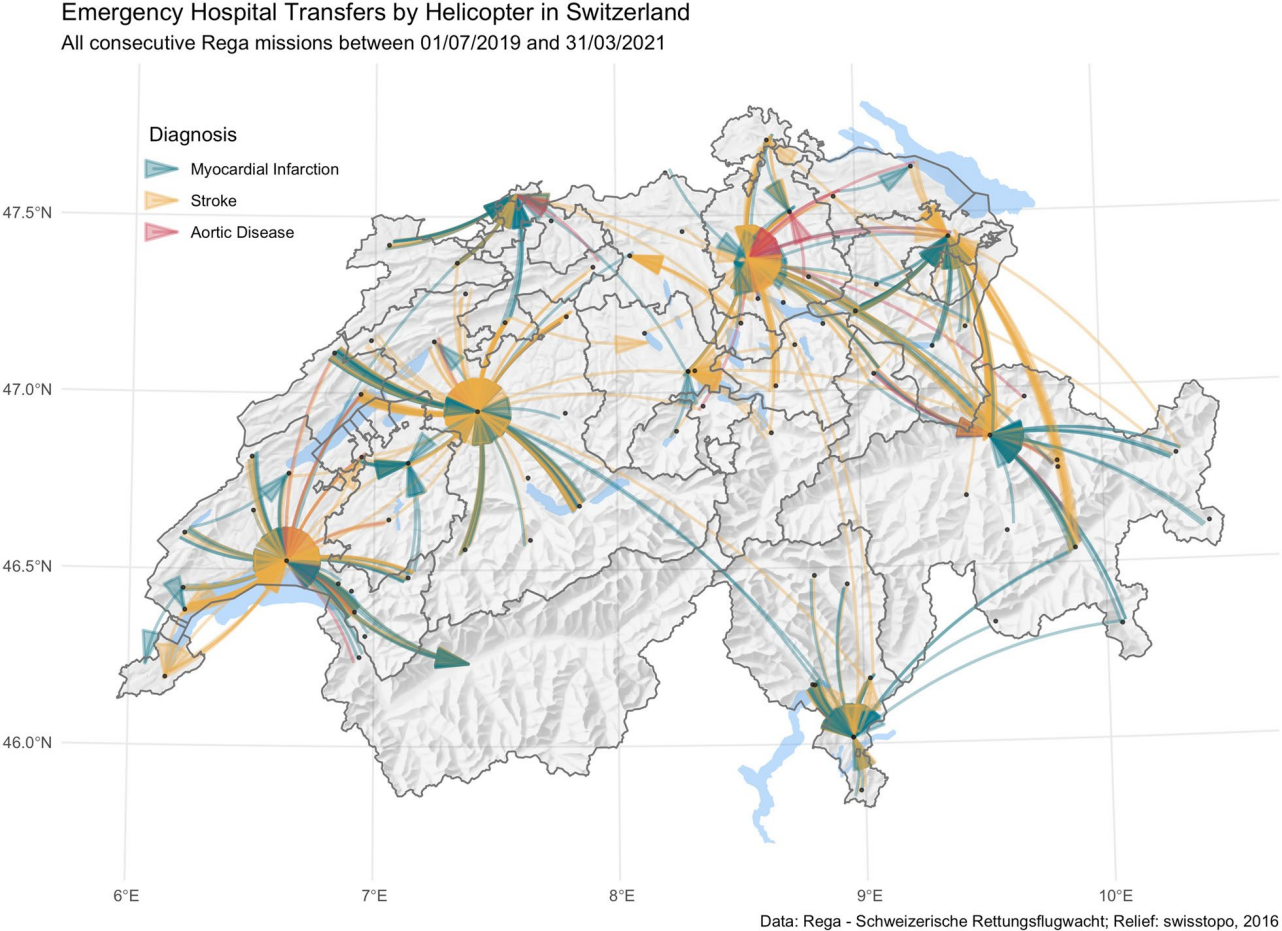
Interfacility transfers for cardiovascular emergencies by HEMS in Switzerland. Topographical map of Switzerland showing cantonal borders and all conducted emergency secondary missions from July 3rd, 2019, and March 31st, 2021. Geo positions of the referral hospitals are jittered to visualize the different numbers of missions per route. Source for relief: swisstopo, 2016

Patients were transferred from 80 institutions, including hospitals and rehabilitation centres, to 26 hospitals and one specialty clinic. 20 level I or II hospitals received 94.0% (n = 606 of 645), see Table 1 and Fig. 1. The nine largest centres received 91.9% (n = 593 of 645) of all transferred patients. Of note, almost half of the patients were referred from level II hospitals to university hospitals (level I hospitals).

**Table 1:** Baseline characteristics of patients and mission details

	Cerebral n = 364	Cardiac n = 252	Vascular n = 29
Age, *years*	69.5 (56.8 to 78.0)	65.0 (55.0 to 75.0)	73.0 (64.0 to 79.0)
Male sex	202 (55.5)	190 (75.4)	26 (89.7)
NACA score	4 (4 to 5)	4 (4 to 5)	5 (4 to 5)
Intubated	72 (19.8)	23 (9.1)	2 (6.9)
Origin			
Level I university hospital (n = 3)	9 (2.5)	2 (0.8)	0
Level II major hospital (n = 44)	185 (50.8)	110 (43.7)	16 (55.2)
Level III regional hospital (n = 10)	70 (19.2)	36 (14.3)	3 (10.3)
Level IV regional hospital (n = 12)	78 (21.4)	72 (28.6)	10 (34.5)
Level V regional hospital (n = 10)	21 (5.8)	32 (12.7)	0
Special clinics (n = 1)	1 (0.3)	0	0
Destination			
Level I university hospital (n = 5)	241 (66.2)	134 (53.2)	23 (79.3)
Level II major hospital (n = 15)	118 (32.4)	83 (32.9)	6 (20.7)
Level III regional hospital (n = 3)	2 (0.5)	2 (0.8)	0
Level IV regional hospital (n = 3)	2 (0.5)	32 (12.7)	0
Level V regional hospital (n = 1)	0	1 (0.4)	0
Specialty clinics (n = 1)	1 (0.3)	0	0
Response time, *min*	22.8 (18.1 to 29.8)	21.7 (16.7 to 27.2)	20.6 (15.2 to 27.5)
Missing	1 (0.3)	2 (0.8)	0
Total mission time, min	59.6 (50.9 to 72.9)	60.8 (52.0 to 70.6)	57.6 (53.0 to 67.2)
Missing	4 (1.1)	2 (0.8)	0
Distance HEMS, *min*	37.8 (29.3 to 48.5)	37.5 (26.8 to 43.6)	38.1 (28.2 to 44.2)
Distance GEMS, *min*	60.0 (42.0 to 69.6)	58.4 (44.7 to 72.9)	52.0 (43.9 to 69.2)

Data was complete if not otherwise stated. Continuous variables were skewed and therefore presented with median and (interquartile range). Counts are presented with percentages in (parentheses). The numbers in parentheses per level for the Origin and Destination variables represent the number of institutions in this level

NACA = National Advisory Committee for Aeronautics, presented by median and interquartile range. Response time = Duration from emergency call to landing at referral hospital. Total mission time = Duration from emergency call to landing at destination hospital. Distance HEMS = Air-line distance for Helicopter Emergency Medical Service. Distance GEMS = Route distance for Ground Emergency Medical Service

None of the 645 patients died during the transfer. Information on in-hospital outcomes is missing.

### Mission duration and travel distance

**Fig. 2:**
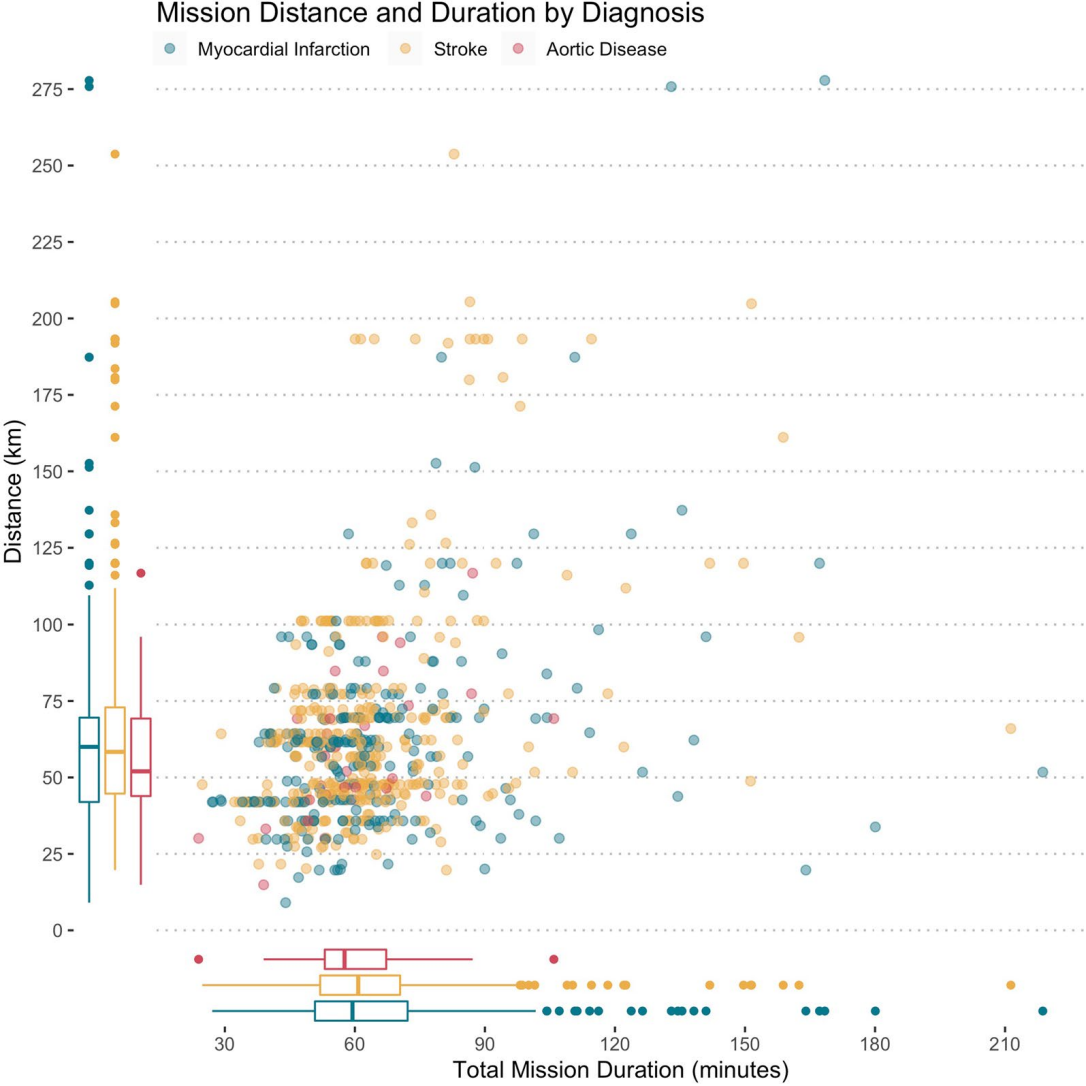
Mission distance and duration by diagnosis. Duration of all conducted HEMS missions is plotted against the travel distance for the same mission by ambulance (road distance). The box plots summarise the total mission duration and road distance by diagnosis, respectively. Complete case analysis, data was available in 639 of 645 patients (99.1%)

The median (interquartile range) total mission time was 59.9 (51.5 to 70.5) minutes. (The median response time was 22.1 (17.1 to 28.1) minutes. The median mission distance for ground-based transportation of the same patient was 60 (42.7 to 72.2) km. Mission duration and travel distances were similar for patients with myocardial infarctions, strokes, or vascular emergencies (see Table 1 and Fig. 2).

Table 2 shows mission details categorised by the road distance between the referring institutions and the destination hospitals. Of note, most HEMS IFT were conducted over relatively short road distances, with 76.7% (495 of 645) of all missions involving a road distance of < 75 km between the two facilities. Short-haul transfers (< 25 km road distance) would have been faster in by ambulance in 41.4% of cases, provided the roads were free and the ambulance was ready to go within 10 min.

**Table 2:** Duration of mission by mode of transportation and ground distance

	0–25 km N = 16	25–50 km N = 243	50–75 km N = 236	75–100 km N = 69	> 100 km N = 81
Duration HEMS, *min*	55.7 (46.3 to 65.6)	57.4 (48.6 to 66.2)	59.5 (51.5 to 67.0)	61.5 (54.0 to 75.9)	77.5 (62.6 to 90.7)
Missing	0	4 (1.7)	2 (0.9)	0	0
Duration GEMS, *min*	34.8 (33.4 to 34.9)	45.6 (38.2 to 51.2)	63.8 (57.5 to 67.8)	77.5 (70.0 to 84.5)	92.6 (70.1 to 148.0)
Difference, *min HEMS—GEMS*	20.2 (10.8 to 33.2)	11.4 (1.3 to 21.5)	− 4.2 (− 10.7 to 27.3)	− 20.0 (− 30.3 to − 8.9)	− 16.8 (− 42.8 to − 5.3)
Missing	0	4 (1.7)	2 (0.9)	0	0

Data was complete if not otherwise stated. Duration of missions are presented in median minutes with (interquartile range)

HEMS = Helicopter Emergency Medical Service. GEMS = Ground Emergency Medical Service. Difference (HEMS—GEMS) = Difference in duration of transportation between HEMS and GEMS for the same missions. A negative number indicates that HEMS is expected to be faster than GEMS

**Fig. 3:**
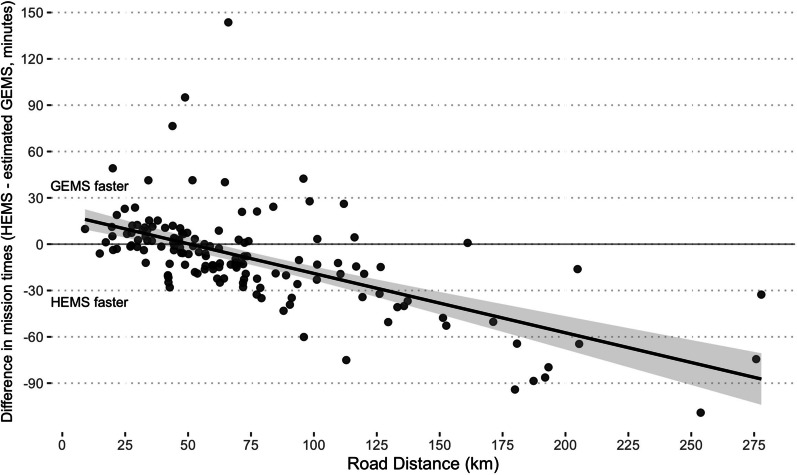
Difference in mission times (HEMS—GEMS). Duration of all conducted Helicopter Emergency Medical Service (HEMS) missions is plotted against the travel distance for the same mission by ambulance (ground-based distance). The black line with grey shading shows the univariate linear regression model with 95% confidence interval. A negative number indicates that HEMS is expected to be faster than GEMS. Complete case analysis, data was available for 639 of 645 patients (99.1%)

Figure 3 shows the differences in travel durations of all HEMS missions and the estimated travel duration for a ground-based transfer based on road distance. The regression analysis revealed that HEMS is expected to be faster if the road distance is more than 51.3 km.

## Discussion

In Switzerland, HEMS enables fast interfacility transfer of patients with cardiovascular emergencies. Most patients are transferred within one hour, and 75% of all patients are transferred within 70.5 min. Road distances of greater than 50 km *and* an estimated total mission duration of one hour or more are generally covered faster by HEMS.

Clinical practice guidelines for the treatment of patients with acute cardiovascular and cerebrovascular diseases recommend the centralisation of patients in specialised centres [7–9, 25]. For the treatment of strokes and myocardial infarctions, patients should be referred to centres where prompt endovascular recanalisation can be attempted [7, 8]. Centralisation should thereby improve treatment results by offering patients the best treatment options and by increasing hospital volume [^2–4,10,26^]. Several studies have documented that it is safe to transfer patients with rAAA to the nearest specialised high-volume vascular center. In fact, such a policy may decrease mortality of the treated patients [25–27]. However, maximum total mission duration for such a transfer has not been proposed. A population-based analysis of patients treated for rAAA in the states of Florida, California, and New York showed that the transfer was associated with lower rates of in-hospital mortality [28]. However, the reduced mortality in the latter study was overshadowed by the proportion of patients who died during transfer without treatment. Furthermore, transferred patients used significantly more hospital resources. The median travel distance reported in this paper was 44.7 km. Unfortunately, information on the duration of the transfer or the transport vehicle used was not reported.

When centralisation of specialised medical services is promoted to increase hospital volume and thus improve treatment results, it is important to provide a rescue chain with emergency medical service that ensures prompt IFT.

Our study demonstrates that HEMS in Switzerland can conduct fast transfers of critically ill patients. A highly specialised and experienced HEMS crew is available 24/7, with a median response time of 22.1 min. Critically ill patients were transferred to specialised centres within a median total mission duration of one hour, and emergency critical care treatments were available en route.

These are very positive findings. However, for most of the conducted missions, road transfers by ambulance would have been equally fast or even faster than HEMS—provided the roads were free and the ambulance was ready to go within 10 min. For patients in many regional hospitals, either GEMS is not available, or the crews do not include a specialised physician, or local regulations do not allow long-distance inter-regional transfers. The presented comparison of helicopter transfers with ambulance transfers assumes that an ambulance and crew are available and ready to go within 10 min of the time of the emergency call to Rega—an assumption which is not met in many situations.

In the Swiss Alps, road detours and closed mountain passes can be expected to extend ground-based travel times. The advantage of HEMS transportation is likely to be most pronounced in the mountainous regions in the southern and eastern parts of Switzerland. The mountainous Canton of Valais (southwest) is served by another HEMS provider and is not covered in this study.

Several factors should be considered when patients need the fastest possible hospital transfer. Hawilo and Taneja named local weather conditions, the severity and urgency of the patient’s illness, the availability of trained personnel, the amount of time for local HEMS dispatch, as well as the likely duration of travel time [29]. We showed that in Switzerland, HEMS is expected to be faster for ground-based travel times of more than one hour. HEMS thereby supports the centralisation of specialised medical treatments in Switzerland by keeping transfer times low even for rural areas in challenging mountainous terrain where ground-based travel times are long.

In cardiovascular emergencies the time to first medical contact (FMC) but also the time from FMC to diagnosis and treatment should be minimized [8]. The European Society of Cardiology recommends a maximum target delay of 10 min from FMC to 12-lead ECG recording and interpretation in patients with persistent chest pain [8]. The working diagnosis of a myocardial infarction should then lead to a direct referral to a specialized hospital offering 24/7 coronary revascularization [8]. Thus, a "load and go" strategy with referral to a smaller hospital without diagnosis might delay the time to treatment even if IFT was conducted fast. Local delays for diagnostics and treatment delays at specialized hospitals should be minimized. This study does *not* cover the entire rescue chain but only IFT in cardiovascular emergencies. Hence, evaluation of the entire rescue chain in Switzerland is needed to assess and finally minimize the time to treatment in patients with cardiovascular emergencies.

The findings of this study might be applicable to other countries where interfacility transfers are affected by topographical and/or infrastructural challenges and provides facts for a critical discussion of the centralisation of patients with time-sensitive cardiovascular emergencies.

This study has several limitations. First, the travel times for road transfers were estimated. No data was available that allows direct comparison for emergency IFTs conducted by GEMS and HEMS. Duration of travel might be faster in good conditions or significantly slower in difficult weather, traffic jams, or road blockages. Further research should focus on a direct and fair comparison of defined benchmark routes. Second, patients’ treatment outcomes are not known. The delay in treatment caused by transfer to a larger medical center might be associated with morbidity that is not assessed in this study. Further, information on the pre-hospital phase was not available. Third, information on the diagnosis and urgency of the 366 declined transfers are missing. Assuming that the proportion of medical emergencies was the same for those missions (i.e.: 15.1%), roughly 55 patients with medical emergencies could not be transferred by HEMS in the observed time period.

## Conclusion

This study demonstrates that HEMS in Switzerland ensures time-sensitive interfacility transfers in medical emergencies, even in topographically challenging terrain. Centralisation of specialised medical services should be accompanied by a comprehensive and specialised rescue chain. However, further research to assess the impact of delayed treatment on outcomes is needed.

## Data Availability

The anonymized data can be made available on request.

## Abbreviations

rAAA: Ruptured abdominal aortic aneurysms
HEMS: Helicopter emergency medical services
IFT: Interfacility patient transfer
ICD: International classification of diseases
IQR: Interquartile range
FMC: First medical contact

## References

[1] Luft HS, Bunker JP, Enthoven AC (1979). Should operations be regionalized?. New Engl J Med..

[2] Vonlanthen R, Lodge P, Barkun JS, Farges O, Rogiers X, Soreide K, et al. Toward a consensus on centralization in surgery. Ann Surg. 2018;268. 10.1097/SLA.0000000000002965 30169394

[3] Güller U, Warschkow R, Ackermann C, Schmied B, Cerny T, Ess S. Lower hospital volume is associated with higher mortality after oesophageal, gastric, pancreatic and rectal cancer resection. Swiss Med Week. 2017;147. 10.4414/smw.2017.14473 28750418

[4] Trenner M, Salvermoser M, Busch A, Schmid V, Eckstein H-H, Kühnl A. The effects of minimum caseload requirements on management and outcome in abdominal aortic aneurysm repair. Deutsches Aerzteblatt Online. 2020; 10.3238/arztebl.2020.0820 PMC8005841 33568259

[5] Scali ST, Beck A, Sedrakyan A, Mao J, Behrendt C-A, Boyle JR, et al. Editor’s choice – optimal threshold for the volume–outcome relationship after open AAA repair in the endovascular era: analysis of the international consortium of vascular registries. Eur J Vasc Endovasc Surg. 2021;61. 10.1016/j.ejvs.2021.02.018 33722485

[6] Scali ST, Beck A, Sedrakyan A, Mao J, Behrendt C-A, Boyle JR, et al. Optimal threshold for the volume–outcome relationship after open AAA repair in the endovascular era: analysis of the international consortium of vascular registries. Eur J Vasc Endovasc Surg. 2021; 10.1016/j.ejvs.2021.02.018 33722485

[7] Powers WJ, Rabinstein AA, Ackerson T, Adeoye OM, Bambakidis NC, Becker K (2018). Guidelines for the early management of patients with acute ischemic stroke: a guideline for healthcare professionals from the American heart association/American stroke association. Stroke.

[8] Ibanez B, James S, Agewall S, Antunes MJ, Bucciarelli-Ducci C, Bueno H (2017). ESC Guidelines for the management of acute myocardial infarction in patients presenting with ST-segment elevation. Eur Heart J.

[9] Björck M, Earnshaw JJ, Acosta S, Bastos Gonçalves F, Cochennec F, Debus ES, et al. Editor’s Choice – European Society for Vascular Surgery (ESVS) 2020 Clinical Practice Guidelines on the Management of Acute Limb Ischaemia. Eur J Vasc Endovasc Surg. 2020;59. 10.1016/j.ejvs.2019.09.006 31899099

[10] Powell JT, Hinchliffe RJ, Thompson MM, Sweeting MJ, Ashleigh R, Bell R, et al. Observations from the IMPROVE trial concerning the clinical care of patients with ruptured abdominal aortic aneurysm. Brit J Surg. 2014;101. 10.1002/bjs.9410 PMC4164272 24469620

[11] Hinchliffe RJ, Ribbons T, Ulug P, Powell JT. Transfer of patients with ruptured abdominal aortic aneurysm from general hospitals to specialist vascular centres: results of a Delphi consensus study: Table 1. Emerg Med J. 2013;30. 10.1136/emermed-2012-201239 PMC3664393 22761515

[12] O’Gara PT, Kushner FG, Ascheim DD, Casey DE, Chung MK, de Lemos JA (2013). ACCF/AHA Guideline for the Management of ST-Elevation Myocardial Infarction. J Am Coll Cardiol.

[13] Boyd CR, Corse KM, CampbellL RC. Emergency interhospital transport of the major trauma patient. J Trauma Injury Infection Crit Care. 1989;29. 10.1097/00005373-198906000-00015 2738976

[14] Arfken CL, Shapiro MJ, Bessey PQ, Littenberg B. Effectiveness of helicopter versus ground ambulance services for interfacility transport. J Trauma Injury Infection Critical Care. 1998;45. 10.1097/00005373-199810000-00031 9783622

[15] Hill AD, Fowler RA, Nathens AB. Impact of interhospital transfer on outcomes for trauma patients: a systematic review. J Trauma Injury, Infection Crit Care. 2011;71. 10.1097/TA.0b013e31823ac642 22182900

[16] Ryb GE, Dischinger P, Cooper C, Kufera JA. Does helicopter transport improve outcomes independently of emergency medical system time? J Trauma Acute Care Surg. 2013;74. 10.1097/TA.0b013e31827890cc 23271090

[17] Foster NA, Elfenbein DM, Kelley W, Brown CR, Foley C, Scarborough JE, et al. Comparison of helicopter versus ground transport for the interfacility transport of isolated spinal injury. Spine Journal. 2014;14. 10.1016/j.spinee.2013.07.478 24139232

[18] Karanicolas PJ, Bhatia P, Williamson J, Malthaner RA, Parry NG, Girotti MJ, et al. The fastest route between two points is not always a straight line: an analysis of air and land transfer of nonpenetrating trauma patients. J Trauma Injury Infect Crit Care. 2006;61. 10.1097/01.ta.0000222974.31728.2a 16917457

[19] Svenson JE, O’Connor JE, Lindsay MB. Is air transport faster? A comparison of air versus ground transport times for interfacility transfers in a regional referral system. Air Med J. 2006;25. 10.1016/j.amj.2006.04.003 16818167

[20] Federal Office of Topography swisstopo [Internet]. [cited 2021 May 12]. Available from: swisstopo.admin.ch

[21] von Elm E, Altman DG, Egger M, Pocock SJ, Gøtzsche PC, Vandenbroucke JP. The Strengthening the Reporting of Observational Studies in Epidemiology (STROBE) statement: guidelines for reporting observational studies. The Lancet. 2007;370. 10.1136/bmj.39335.541782.AD PMC2034723 17947786

[22] Statistik der stationären Betriebe des Gesundheitswesens. Krankenhaustypologie [Internet]. Statistik der stationären Betriebe des Gesundheitswesens. Krankenhaustypologie. 2013. Available from: https://www.bfs.admin.ch/hub/api/dam/assets/169879/master

[23] Melo R, Zarruk D. gmapsdistance - Distance and travel time between two points from google maps. 2016.

[24] R Core Team (2013). R: A language and environment for statistical computing. R Foundation for Statistical Computing, Vienna, Austria. URL http://www.R-project.org/. RStudio, PBC, Boston, MA;

[25] Wanhainen A, Verzini F, van Herzeele I, Allaire E, Bown M, Cohnert T, et al. Editor’s Choice – European Society for Vascular Surgery (ESVS) 2019 Clinical Practice Guidelines on the Management of Abdominal Aorto-iliac Artery Aneurysms. Eur J Vasc Endovasc Surg. W.B. Saunders Ltd; 2019;57:8–93. 10.1016/j.ejvs.2018.09.020 30528142

[26] Park BD, Azefor N, Huang C-C, Ricotta JJ. Trends in treatment of ruptured abdominal aortic aneurysm: impact of endovascular repair and implications for future care. J Am Coll Surg. 2013;216. 10.1016/j.jamcollsurg.2012.12.028 23521956

[27] Mandawat A, Mandawat A, Sosa JA, Muhs BE, Indes JE. Endovascular repair is associated with superior clinical outcomes in patients transferred for treatment of ruptured abdominal aortic aneurysms. J Endovasc Therapy. 2012;19. 10.1583/11-3651.1 22313208

[28] Mell MW, Wang NE, Morrison DE, Hernandez-Boussard T. Interfacility transfer and mortality for patients with ruptured abdominal aortic aneurysm. J Vasc Surg. 2014;60. 10.1016/j.jvs.2014.02.061 24768368

[29] Hawilo H, Taneja R. Interfacility helicopter transfers for critically ill patients: always the right choice? Critical Care. 2020;24. 10.1186/s13054-020-02846-1 PMC7164246 32299474

